# Pediatric Autoimmune Neuropsychiatric Disorders Associated with Streptococcal Infections (PANDAS): Autoimmune Mechanisms, Diagnostic Controversies, and Therapeutic Implications

**DOI:** 10.26502/jppch.74050244

**Published:** 2026-07-14

**Authors:** Victoria Lynne, Devendra K. Agrawal

**Affiliations:** Department of Translational Research, College of Osteopathic Medicine of the Pacific, Western University of Health Sciences, Pomona, California 91766, USA

**Keywords:** Autoimmune encephalitis, Group A Streptococcus, Molecular mimicry, Neuroinflammation, Obsessive-compulsive disorder, Pediatric Autoimmune Neuropsychiatric Disorders Associated with Streptococcal Infections (PANDAS), Pediatric Acute-Onset Neuropsychiatric Syndrome (PANS), Pediatric neuropsychiatry, Tic disorders

## Abstract

Pediatric Autoimmune Neuropsychiatric Disorders Associated with Streptococcal Infections (PANDAS) is a proposed postinfectious neuroimmune syndrome characterized by the abrupt onset of obsessive-compulsive disorder, tic disorders, and associated neuropsychiatric symptoms temporally associated with Group A beta-hemolytic Streptococcus infection. Since its initial description by Swedo and colleagues in 1998, PANDAS has generated notable scientific interest and controversy. Increasing evidence from immunology, neuroimaging, epidemiology, and translational neuroscience suggests that infection-triggered autoimmune processes may contribute to neuropsychiatric symptom development in a subset of pediatric patients. However, significant uncertainty remains regarding diagnostic validity, biomarker reproducibility, pathophysiologic mechanisms, and optimal treatment strategies. This review carefully evaluates current evidence regarding the epidemiology, immunopathogenesis, clinical manifestations, diagnosis, treatment, prognosis, and controversies surrounding PANDAS and related Pediatric Acute-Onset Neuropsychiatric Syndrome (PANS). Relevant literature published from 1998 through May 2026 was critically reviewed. Recent publications from 2021-2026 were incorporated to update evidence on clinical guidance, neuroimaging, immune biomarkers, autoimmune comorbidity, and IVIG outcomes. Evidence supporting the PANDAS construct includes epidemiologic associations with streptococcal infection, parallels with Sydenham chorea, identification of antineuronal antibodies, neuroimaging abnormalities involving basal ganglia structures, translational animal models, and selective responsiveness to immunomodulatory therapies. Conversely, substantial concerns remain regarding inconsistent biomarker replication, methodological heterogeneity, diagnostic overlap with primary psychiatric disorders, and the absence of universally accepted diagnostic tests. Current evidence supports the biologic plausibility of infection-triggered neuroimmune neuropsychiatric syndromes in a subset of pediatric patients. However, the specificity of the PANDAS construct, the boundaries between PANDAS and Pediatric Acute-Onset Neuropsychiatric Syndrome, and the identification of immune-responsive subgroups remain incompletely defined. Future multicenter prospective studies incorporating standardized diagnostic criteria, advanced immunophenotyping, neuroimaging, genomics, and longitudinal outcome measures are required to clarify disease mechanisms and optimize patient management.

## 1. Introduction

The interaction between infectious disease and neuropsychiatric symptomatology has intrigued clinicians for more than a century. Historical observations linking streptococcal infections to Sydenham chorea [1,2], rheumatic fever, and behavioral disturbances provided early evidence that immune responses to infection can influence central nervous system function. These observations ultimately formed the conceptual foundation for Pediatric Autoimmune Neuropsychiatric Disorders Associated with Streptococcal Infections (PANDAS), a syndrome proposed to explain the abrupt onset of obsessive-compulsive and tic symptoms following streptococcal infection.

PANDAS was formally described in 1998 by Swedo and colleagues [3], who identified children with abrupt onset of obsessive-compulsive disorder (OCD) or tic disorders temporally associated with Group A beta-hemolytic Streptococcus infection (GAS) infection. Unlike conventional childhood OCD, which often develops gradually, reported onset in PANDAS was sudden, sometimes over days or hours, and frequently accompanied by severe anxiety, compulsive rituals, emotional instability, motor abnormalities, and functional decline.

The proposed biologic basis of PANDAS emerged from parallels with Sydenham chorea [1,2], the neurologic manifestation of acute rheumatic fever. In Sydenham chorea, antibodies generated against streptococcal antigens cross-react with host neuronal tissue through molecular mimicry [4,5], resulting in basal ganglia dysfunction and characteristic neurologic manifestations. Investigators hypothesized that a similar autoimmune process might contribute to the abrupt neuropsychiatric symptoms observed in children meeting PANDAS criteria.

Over the subsequent two decades, increasing attention has been directed toward infection-triggered neuroimmune interactions. Research has explored molecular mimicry, antineuronal antibodies, cytokine dysregulation, blood-brain barrier dysfunction, microglial activation, and neuroinflammation as potential contributors to symptom development [6,7]. Simultaneously, substantial controversy has emerged regarding diagnostic validity, causality, and treatment. While some clinicians regard PANDAS as a legitimate autoimmune neuropsychiatric disorder [1,8], others argue that available evidence remains insufficient to distinguish the syndrome from naturally fluctuating psychiatric illness [9].

The debate has been further complicated by the development of Pediatric Acute-Onset Neuropsychiatric Syndrome (PANS), a broader diagnostic framework encompassing acute-onset OCD and related neuropsychiatric symptoms regardless of infectious trigger. This expansion reflects growing recognition that multiple infectious, inflammatory, and possibly autoimmune mechanisms may contribute to abrupt psychiatric deterioration in children [6,7].

Given the implications for psychiatry, neurology, immunology, infectious disease, and pediatrics, this review evaluates the evolution of the PANDAS concept, epidemiology, proposed mechanisms, clinical manifestations, diagnostic approaches, treatment, controversies, and future research priorities.

## 2. Methods

This comprehensive narrative review synthesizes current evidence regarding PANDAS and the broader PANS framework, with emphasis on historical development, epidemiology, immunopathogenesis, clinical presentation, diagnosis, treatment, controversies, and future research priorities. Relevant literature was identified through PubMed/MEDLINE and Google Scholar. Searches covered January 1998 through May 2026 and combined terms related to PANDAS, PANS, pediatric acute-onset OCD, tic disorders, Group A Streptococcus, Sydenham chorea, molecular mimicry, antineuronal antibodies, basal ganglia, neuroinflammation, cytokines, Th17 cells, blood-brain barrier dysfunction, microbiome mechanisms, neuroimaging, antibiotics, corticosteroids, intravenous immunoglobulin, plasma exchange, and immunotherapy.

Since this review was intended to provide a comprehensive overview of the field rather than a formal systematic review or meta-analysis, literature selection emphasized relevance, scientific impact, methodological rigor, and historical importance. Priority was given to landmark PANDAS publications, prospective cohort studies, case-control investigations, randomized controlled trials, translational immunologic research, and case reports. Findings were synthesized narratively. Emphasis was placed on areas of agreement, conflicting evidence, diagnostic controversies, treatment controversies, methodological limitations, and emerging research directions.

### 2.1 Limitations in the Methods

The literature is characterized by substantial heterogeneity in diagnostic criteria, study design, patient selection, laboratory methodology, and outcome assessment. Because this manuscript is a narrative review rather than a formal systematic review, publication selection was not governed by predefined PRISMA criteria and quantitative meta-analysis was not performed. Conclusions should therefore be interpreted within an evolving and sometimes conflicting evidence base.

## 3. Historical Evolution of the PANDAS Concept

The origins of the PANDAS hypothesis are deeply rooted in observations of Sydenham chorea [1,2]. Long recognized as a poststreptococcal autoimmune disorder, Sydenham chorea is characterized by involuntary movements, emotional lability, obsessive-compulsive symptoms, and basal ganglia dysfunction occurring weeks to months after streptococcal infection. The established autoimmune nature of Sydenham chorea provided a biologically plausible framework for considering whether other neuropsychiatric syndromes might similarly arise from aberrant immune responses.

In 1998, Swedo and colleagues [3] proposed five working diagnostic criteria for PANDAS: (i) Presence of OCD and/or tic disorder, (ii) Pediatric onset, (iii) Episodic symptom course, (iv) Temporal relationship with GAS infection, and (v) Neurologic abnormalities including motor hyperactivity or choreiform movements. The original publication generated considerable interest because it suggested that a subset of childhood psychiatric disorders might have a treatable immunologic basis [3].

Subsequent epidemiologic, immunologic, and neuroimaging studies reported associations with streptococcal exposure, antineuronal antibodies, and basal ganglia circuitry abnormalities, but replication has been inconsistent [9,10].

Recognition that acute neuropsychiatric deterioration may follow triggers other than GAS led to the broader PANS framework, introduced in 2012, which emphasizes abrupt-onset OCD or restrictive eating accompanied by additional neuropsychiatric symptoms [11].

The transition from PANDAS to PANS reflects an important conceptual shift. Whereas PANDAS focuses on a specific infectious trigger, PANS emphasizes a broader syndrome potentially arising from multiple infectious, inflammatory, metabolic, or autoimmune mechanisms [8,11]. This evolution highlights growing recognition of the complex relationship between immunity and neuropsychiatric function.

### 3.1 Epidemiology

Determining the true epidemiology of PANDAS remains challenging because of diagnostic uncertainty, evolving definitions, and the absence of validated biomarkers [9-11]. Consequently, estimates of prevalence and incidence vary substantially across studies [9,12].

Available evidence suggests that PANDAS primarily affects children between approximately three years of age and puberty, with peak onset occurring near six to eight years of age [3,8,13]. This age distribution parallels periods of increased streptococcal exposure and ongoing neurodevelopment of cortico-striato-thalamo-cortical circuitry. The apparent restriction to childhood has been cited as indirect support for developmental susceptibility factors, including age-dependent immune responses and neuroplasticity [1].

Most cohorts demonstrate a male predominance, with reported male-to-female ratios ranging from approximately 2:1 to 3:1 [3,13,14]. This sex distribution resembles that observed in childhood-onset OCD and tic disorders but differs from many classical autoimmune diseases, which typically exhibit female predominance. The reasons for this discrepancy remain uncertain but may involve developmental, hormonal, genetic, or neurobiological influences [15].

Familial aggregation studies have identified increased prevalence of autoimmune disorders, OCD, tic disorders, and rheumatic fever among relatives of affected children. Such findings raise the possibility that genetic susceptibility influences both immune regulation and neuropsychiatric vulnerability [16,17]. However, the specific genetic factors underlying this susceptibility remain poorly defined.

More recent cohort data further support the need to distinguish familial autoimmune vulnerability from disease-specific causality. A 2024 cohort study of children with PANS reported evidence of immune activation and subsequent autoimmune disease development in a subset of patients, suggesting that PANS populations may include children with broader inflammatory or autoimmune predisposition rather than a single uniform poststreptococcal mechanism [18].

Several case-control and administrative database studies have reported increased odds of recent GAS exposure among children with OCD or tic disorders, including studies by Mell and Leslie and colleagues [19,20].

Because streptococcal infections and asymptomatic GAS carriage are common in childhood, temporal association alone does not establish causality [9,20,21].

Longitudinal studies are mixed: some report associations between new GAS infections and symptom exacerbations, whereas others do not. Differences in diagnostic criteria, infection ascertainment, outcome measures, and follow-up likely contribute to these discrepancies [19-22].

An additional challenge involves distinguishing active infection from asymptomatic carriage. Positive throat cultures may reflect colonization rather than clinically meaningful infection. Because immune responses rather than bacterial presence are presumed to drive symptom development, accurately characterizing infectious exposure remains difficult [21,23,24].

Several factors may explain the inconsistent epidemiologic findings reported across studies. First, streptococcal infections are extremely common during childhood, increasing the likelihood of coincidental temporal associations. Second, studies differ substantially in their methods of confirming infection, ranging from throat cultures and serologic testing to administrative database coding. Third, diagnostic definitions of PANDAS and PANS vary considerably across investigations, resulting in heterogeneous study populations. Fourth, some studies assess first onset whereas others examine symptom exacerbations, which may represent biologically distinct outcomes. Consequently, epidemiologic evidence is best interpreted as supporting an association between infection and neuropsychiatric symptom exacerbation in a subset of susceptible children rather than establishing a uniform causal relationship across all patients [19-21].

### 3.2 Immunopathogenesis of PANDAS

**Figure 1:**
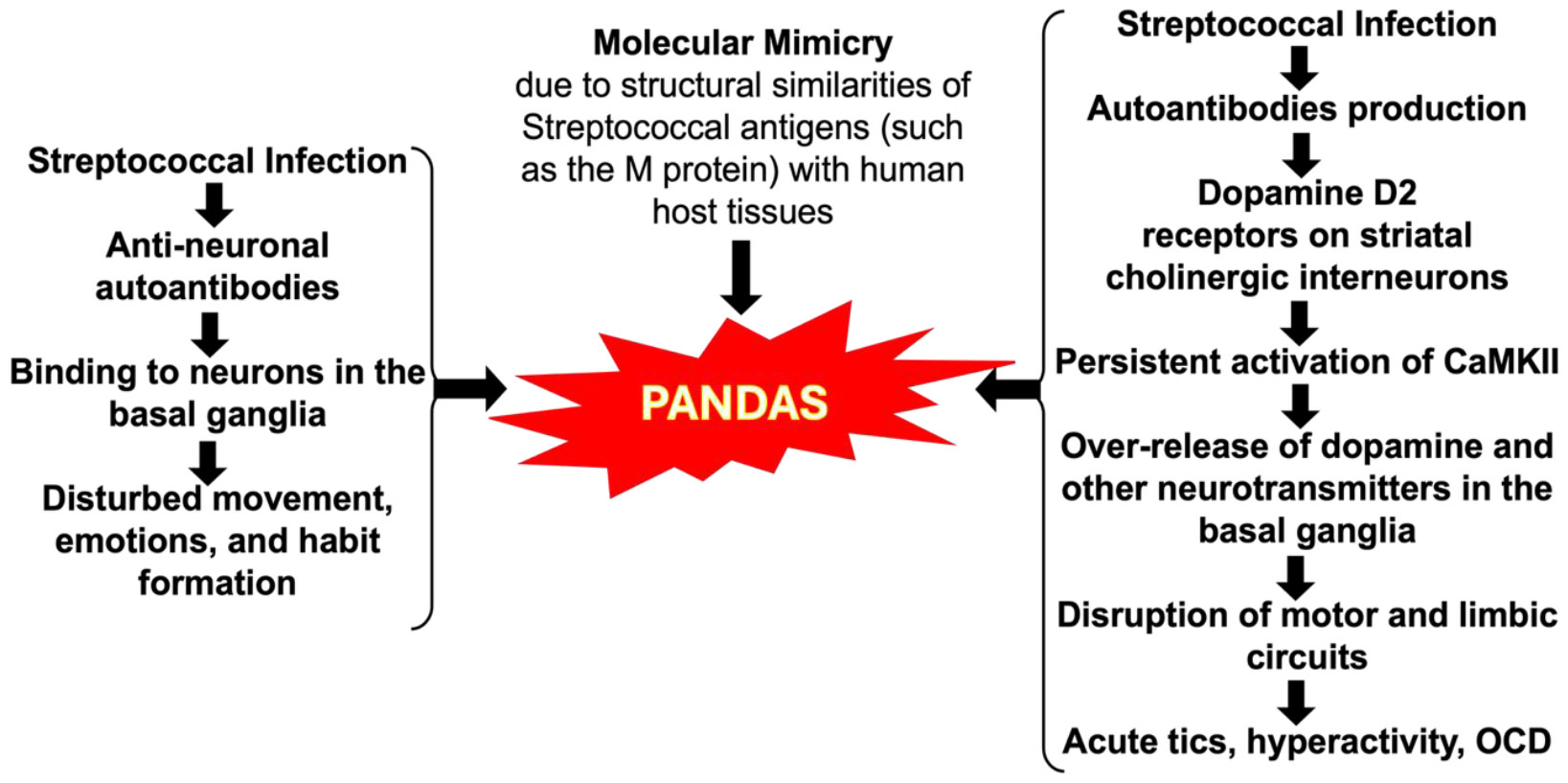
Maladaptive immune response in the underlying pathophysiology of PANDAS: Key mechanistic features. CaMKII, calcium/calmodulin-dependent protein kinase II; OCD, obsessive compulsive disorder; PANDAS, pediatric autoimmune neuropsychiatric disorders associated with streptococcal infections.

The proposed pathophysiology of Pediatric Autoimmune Neuropsychiatric Disorders Associated with Streptococcal Infections (PANDAS) centers on the hypothesis that Group A beta-hemolytic Streptococcus (GAS) infection initiates an aberrant immune response that subsequently targets components of the central nervous system. Although definitive mechanistic proof remains elusive, the conceptual framework underlying PANDAS is supported by decades of research into postinfectious autoimmune diseases, particularly acute rheumatic fever and Sydenham chorea (Figure 1) [1,2].

Unlike conventional psychiatric disorders, which are generally conceptualized within neurodevelopmental, neurochemical, or psychosocial frameworks, PANDAS proposes that immune-mediated disruption of neural circuits contributes directly to symptom emergence. The syndrome therefore occupies a unique position at the intersection of psychiatry, immunology, neurology, and infectious disease. Current models suggest that disease expression results from interactions among infectious exposure, host genetic susceptibility, immune dysregulation, blood-brain barrier dysfunction, and neuroinflammatory processes affecting cortico-striato-thalamo-cortical (CSTC) circuitry [6,7].

The basal ganglia have emerged as a central neuroanatomical target in PANDAS pathophysiology. These structures play critical roles in motor control, habit formation, emotional regulation, reward processing, and executive function. Dysfunction of CSTC pathways has long been implicated in obsessive-compulsive disorder and tic disorders [25,26]. Consequently, autoimmune-mediated disruption of basal ganglia function provides a biologically plausible explanation for the abrupt onset of obsessive-compulsive symptoms, motor abnormalities, emotional lability, and behavioral dysregulation observed in affected children (Figure 1) [1,4,5].

### 3.3 Molecular Mimicry

Molecular mimicry [1,4] remains the leading mechanistic hypothesis. In this process, structural similarities between microbial antigens and host proteins produce immune cross-reactivity, as classically described in acute rheumatic fever. In PANDAS, GAS antigens are proposed to share homology with neuronal proteins in basal ganglia circuits, allowing antibodies generated during infection to cross-react with neuronal targets in susceptible individuals [4,5].

Cunningham and colleagues advanced this hypothesis by identifying cross-reactive antibodies to lysoganglioside GM1, tubulin, and dopamine receptors, suggesting a biologic link between streptococcal immunity and altered neuronal signaling [5].

However, the molecular mimicry hypothesis should not be viewed as definitively established. Although biologically plausible, many studies demonstrating antibody cross-reactivity involve relatively small samples and frequently originate from specialized referral centers. Furthermore, the presence of cross-reactive antibodies does not necessarily prove pathogenicity. Similar antibodies have occasionally been identified in individuals without neuropsychiatric symptoms, raising important questions regarding specificity.

Thus, molecular mimicry likely contributes in some patients but is unlikely to explain all PANDAS/PANS presentations. A key interpretive issue is that molecular mimicry provides biologic plausibility but not diagnostic specificity. Similar postinfectious mechanisms may operate across several neuroimmune disorders, and the presence of cross-reactive antibodies does not by itself demonstrate disease causation. Stronger causal inference will require longitudinal studies showing that specific immune responses precede symptom onset, correlate with disease activity, access relevant neural targets, and diminish with clinical improvement [9,10,12].

### 3.4 Antineuronal Antibodies

One of the most extensively studied aspects of PANDAS involves the identification of antibodies directed against neuronal antigens. These antibodies have been proposed as both mechanistic contributors and potential biomarkers of disease [5,10,27,28].

Reported targets include dopamine D1 and D2 receptors, lysoganglioside GM1, and tubulin, all relevant because of their potential effects on basal ganglia neurotransmission [5,28].

Dopaminergic signaling plays a fundamental role in motor regulation, compulsivity, reward processing, and habit formation. Alterations in dopamine receptor activity have long been implicated in both OCD and tic disorders. Consequently, antibody-mediated interference with dopaminergic pathways represents an attractive mechanistic explanation for many clinical manifestations observed in PANDAS [5,28].

Studies reporting increased calcium/calmodulin-dependent protein kinase II (CaMKII) activation suggested that circulating antibodies might exert functional effects beyond simple antigen recognition [5,10].

Despite initial enthusiasm, reproducibility has proven challenging. Independent studies have yielded mixed results regarding both prevalence and specificity of antineuronal antibodies. Some investigations have demonstrated substantial overlap between presumed PANDAS/PANS populations and healthy controls. Others have failed to replicate previously reported associations [10,29].

These discrepancies highlight a broader challenge within neuroimmunology. Autoantibodies may be pathogenic, epiphenomenal, or unrelated to disease processes. Distinguishing among these possibilities requires rigorous longitudinal studies demonstrating temporal relationships between antibody levels and symptom expression, a standard that remains incompletely achieved within current PANDAS literature [10,29].

Accordingly, although antineuronal antibodies remain among the most promising biologic findings associated with PANDAS, their precise role in disease pathogenesis remains controversial.

The conflicting antibody literature may reflect several nonexclusive possibilities. One possibility is that antineuronal antibodies identify only a biologically distinct subgroup of patients rather than all individuals meeting PANDAS or PANS criteria. Another is that variability in assay methodology, timing of specimen collection, disease activity, and patient selection contributes substantially to inconsistent results. It is also possible that currently studied antibodies function primarily as markers of immune activation rather than direct mediators of disease. These considerations highlight the need for future studies incorporating standardized laboratory methods, longitudinal sampling, and biologically defined patient subgroups [10,29].

Newer antibody work has also shifted attention from broad anti-basal-ganglia reactivity toward more specific neuronal-cell targets. Studies of PANDAS/PANS plasma have reported antibody binding to striatal cholinergic interneurons, a finding that is mechanistically relevant because these interneurons modulate basal ganglia output and may influence motor, habit, and compulsivity circuits. These results are promising but should be interpreted as subgroup-level mechanistic evidence rather than a validated diagnostic biomarker [30].

### 3.5 Evidence Supporting an Autoimmune Etiology

**Figure 2:**
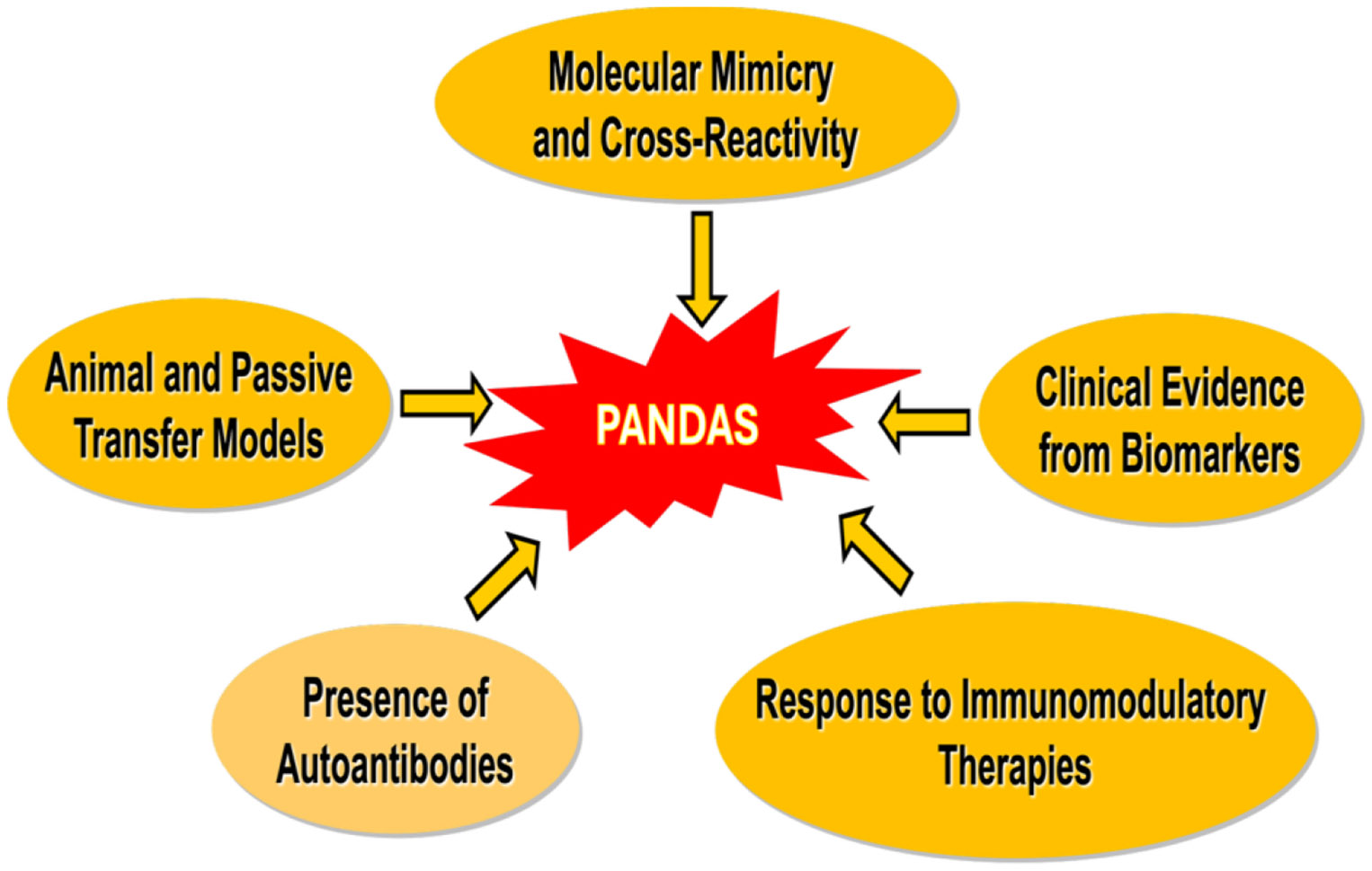
Key Pillars of the autoimmune framework of PANDAS (pediatric autoimmune neuropsychiatric disorders associated with streptococcal infections).

Several converging lines of evidence support immune-mediated mechanisms in at least a subset of PANDAS cases (Figure 2) [1,8,31].

Fourth, biomarkers and neuroimaging studies have reported abnormalities involving basal ganglia structures and inflammatory signaling pathways [32-34].

First, substantial clinical overlap exists between PANDAS and Sydenham chorea [1,2], a well-established poststreptococcal autoimmune disorder. Similarities include abrupt symptom onset, emotional lability, obsessive-compulsive features, motor abnormalities, and basal ganglia involvement.

Second, antineuronal antibodies have been identified in multiple cohorts and have demonstrated functional effects in experimental systems [5,27,28].

Third, animal models have shown that transfer of human antibodies can induce behavioral and dopaminergic abnormalities resembling aspects of the human condition [28].

Fifth, selective responsiveness to immunomodulatory therapies—including corticosteroids, intravenous immunoglobulin (IVIG), and plasma exchange—suggests that immune mechanisms may contribute to symptom generation in at least some patients [35-39].

Importantly, no single line of evidence independently proves the autoimmune hypothesis. However, taken together, these findings create a biologically coherent framework supporting the existence of infection-triggered neuroimmune neuropsychiatric syndromes [1,8,31].

At the same time, substantial methodological limitations remain, and many findings require replication in larger prospective cohorts [10,40]. Therefore, the most scientifically defensible conclusion is not that PANDAS has been definitively proven or disproven, but rather that growing evidence supports immune-mediated mechanisms in a subset of affected children while significant uncertainties also remain [10,40].

### 3.6 Evidence Challenging the Autoimmune Hypothesis

Although substantial evidence supports immune-mediated mechanisms in at least some children with acute-onset neuropsychiatric symptoms, important findings challenge the notion that PANDAS represents a distinct, uniformly autoimmune disorder. Indeed, the controversy surrounding PANDAS largely stems not from the absence of biologic plausibility, but rather from inconsistencies in epidemiologic, immunologic, and clinical evidence [10,40].

A major challenge in evaluating PANDAS involves distinguishing biologic association from causation. Many of the observations supporting the syndrome—including positive streptococcal cultures, elevated antistreptococcal antibody titers, and detection of antineuronal antibodies—are not unique to affected patients. Because exposure to Group A Streptococcus is extremely common during childhood, many healthy children possess elevated antibody titers or evidence of prior infection. Consequently, the mere presence of streptococcal exposure does not establish a causal role in neuropsychiatric symptom development [9,20,21].

Furthermore, several prospective investigations have failed to demonstrate a consistent temporal relationship between new streptococcal infections and symptom exacerbations. While some cohorts report symptom worsening following documented infections, others have observed no meaningful correlation between GAS exposure and subsequent neuropsychiatric deterioration. These inconsistencies raise the possibility that streptococcal infection may function as one of many nonspecific stressors capable of influencing symptom expression rather than serving as a unique etiologic trigger [19,20,21].

The heterogeneity of reported clinical presentations further complicates interpretation. Some children meeting PANDAS criteria exhibit dramatic symptom onset, recurrent exacerbations, and apparent responsiveness to immunomodulatory interventions. Others demonstrate symptom trajectories nearly indistinguishable from conventional obsessive-compulsive disorder or tic disorders. This variability suggests that current diagnostic criteria may encompass multiple biologically distinct conditions rather than a single disease entity [9,11,41].

### 3.7 Basal Ganglia Dysfunction

The basal ganglia occupy a central position in contemporary models of PANDAS pathophysiology. These interconnected nuclei modulate motor control, cognitive flexibility, emotional regulation, procedural learning, and habit formation. Dysfunction within CSTC circuitry has consistently been implicated in both OCD and tic disorders [25,26].

The hypothesis that immune-mediated injury to basal ganglia structures contributes to symptom development derives largely from parallels with Sydenham chorea [1,2]. Patients with Sydenham chorea exhibit abnormalities involving the caudate nucleus, putamen, and globus pallidus, regions similarly implicated in OCD pathophysiology.

Neuroimaging studies have provided preliminary support for this model. Structural imaging investigations have reported enlargement of basal ganglia structures in some children with acute-onset neuropsychiatric symptoms. Functional imaging studies have additionally demonstrated abnormalities involving metabolism, inflammation, and connectivity within CSTC networks [32,33].

More recent diffusion-weighted MRI work has expanded this neuroimaging literature by identifying microstructural differences across multiple brain regions in children with PANS, including deep gray matter structures. These findings strengthen the rationale for studying network-level and inflammatory imaging biomarkers, but they remain insufficient for individual diagnosis because imaging abnormalities are not yet standardized, specific, or prospectively validated [34].

Nevertheless, neuroimaging findings remain inconsistent. Many patients demonstrate normal magnetic resonance imaging, and observed abnormalities often fail to replicate across independent cohorts. Small sample sizes and methodological heterogeneity further limit interpretation [32-34].

Taken together, contemporary neuroimaging findings are best viewed as mechanistic rather than diagnostic evidence. The observed abnormalities support involvement of basal ganglia and distributed cortico-striato-thalamo-cortical networks, but no imaging pattern has demonstrated sufficient specificity to distinguish PANDAS/PANS from primary OCD, tic disorders, Sydenham chorea, autoimmune encephalitis, or other pediatric neuropsychiatric conditions. Future longitudinal imaging studies integrating immune biomarkers, symptom trajectories, treatment response, and matched psychiatric controls may prove more informative than structural imaging alone [32-34].

### 3.8 Blood-Brain Barrier Dysfunction

A critical question is how circulating antibodies or immune mediators access neural tissue, given that the blood-brain barrier (BBB) normally restricts entry of large molecules into the central nervous system [7,26].

Systemic inflammation may transiently alter BBB permeability, facilitate immune-cell migration and allow antineuronal antibodies to access vulnerable basal ganglia circuits [7,26].

Animal studies have provided important mechanistic support. Investigations involving intranasal GAS infection have demonstrated migration of streptococcal-specific immune cells into central nervous system tissues. These findings suggest that infection-induced inflammatory responses may facilitate immune access to neural structures [7].

Although translational findings are compelling, direct evidence of BBB dysfunction in human PANDAS populations remains limited. Future studies utilizing advanced neuroimaging and cerebrospinal fluid analyses may help clarify the role of barrier integrity in disease development.

### 3.9 Cytokine Dysregulation and Neuroinflammation

Neuroinflammatory processes, particularly cytokine-mediated signaling, may contribute to symptom development in some patients [6,7,26].

Several investigations have reported elevated concentrations of proinflammatory cytokines, including tumor necrosis factor-α (TNF-α), interleukin-6 (IL-6), and interleukin-17 (IL-17), among subsets of children meeting PANS/PANDAS criteria. These molecules influence immune-cell trafficking, BBB permeability, neurotransmission, and neuronal survival [6,26].

Attention has focused on Th17 lymphocytes. Originally characterized in autoimmune diseases such as multiple sclerosis, Th17 cells produce IL-17 and promote inflammatory responses. Experimental studies suggest that streptococcal infections can induce Th17-mediated immune activation capable of influencing central nervous system function [7,26].

Neuroinflammation may contribute to symptom development through multiple mechanisms. Cytokines influence dopamine synthesis, glutamatergic transmission, synaptic plasticity, and microglial activation. Collectively, these effects may alter neural network activity and contribute to abrupt neuropsychiatric deterioration [6,7,26].

Importantly, neuroinflammation does not necessarily imply classical autoimmune disease. Similar inflammatory pathways have been implicated across a broad spectrum of neurologic and psychiatric disorders. Consequently, inflammatory findings should be viewed as supportive rather than definitive evidence for PANDAS-specific mechanisms [6,26].

The inflammatory literature therefore requires careful interpretation. Cytokine abnormalities may reflect causal mechanisms, downstream consequences of severe psychiatric stress, intercurrent infection, medication exposure, or nonspecific immune activation. Studies that collect samples during acute flares, remission, and matched infectious episodes in controls will be particularly important for distinguishing state markers from disease mechanisms [6,26].

### 3.10 Gut-Brain Axis and Microbiome Research

An emerging area of investigation involves interactions among the gut microbiome, immune regulation, and neuropsychiatric function. Increasing evidence suggests that intestinal microbial communities influence immune development, cytokine production, and neurochemical signaling [42].

Preliminary studies have reported alterations in gut microbiota composition among children with PANS and PANDAS. Although findings remain exploratory, investigators have proposed that dysbiosis may contribute to immune dysregulation, systemic inflammation, and altered neuroimmune communication [42].

The gut-brain axis represents a rapidly evolving area of neuroscience. However, current microbiome evidence remains insufficient to establish causal relationships. Future longitudinal studies integrating microbiome sequencing, metabolomics, immunophenotyping, and clinical outcomes may help clarify whether microbial alterations contribute meaningfully to disease pathogenesis [42].

## 4. Clinical Manifestations

The clinical phenotype described in PANDAS and PANS is broad, but its most distinctive feature is abrupt onset [3,8,11]. Children may develop obsessive-compulsive symptoms, tics, restrictive eating, separation anxiety, emotional lability, irritability, sleep disturbance, urinary frequency, sensory sensitivity, handwriting deterioration, or academic decline over days rather than months. This acute trajectory distinguishes the proposed syndrome from many typical presentations of pediatric OCD or tic disorders, although overlap remains substantial [9].

Abrupt-onset OCD remains the core manifestation. Reported obsessions commonly include contamination fears, intrusive harm-related thoughts, excessive doubt, and reassurance seeking, whereas compulsions may include washing, checking, counting, ritualized questioning, and avoidance. Tics may be motor or vocal and can range from simple eye blinking or throat clearing to more complex movements or vocalizations. Choreiform movements, fine motor deterioration, and handwriting regression are particularly relevant because they reinforce comparisons with Sydenham chorea [1,2].

Associated behavioral and somatic symptoms often drive impairment. Separation anxiety, panic-like symptoms, mood lability, rage episodes, sleep disruption, urinary urgency, sensory sensitivity, headaches, fatigue, and gastrointestinal complaints have all been reported [8,11,43]. Cognitive and academic changes, including reduced concentration, slowed processing, executive dysfunction, and sudden decline in school performance, may be especially disruptive for previously high-functioning children [8,43].

The breadth of symptoms supports the possibility of distributed network dysfunction, but it also reduces diagnostic specificity. Many associated features are common across pediatric psychiatric, neurodevelopmental, and functional disorders. Therefore, symptoms should not be interpreted in isolation. Abrupt onset, episodic course, neurologic findings, documented infectious exposure, evidence of immune activation, and longitudinal pattern are more informative when considered together [44,45].

### 4.1 Diagnostic Evaluation

The diagnosis of PANDAS remains fundamentally clinical because no laboratory assay, imaging study, or biomarker has demonstrated sufficient sensitivity and specificity to independently establish or exclude the diagnosis [44-46]. Consequently, clinicians must integrate historical features, physical examination findings, laboratory studies, psychiatric assessment, and exclusion of alternative disorders when evaluating suspected cases.

A defining characteristic of PANDAS is the abrupt onset of obsessive-compulsive symptoms and/or tic disorders. Obtaining a detailed chronology of symptom development is therefore critical. Parents frequently report a dramatic behavioral transformation occurring over hours to days rather than the gradual progression more typical of conventional childhood OCD [3,8]. Documentation of symptom onset, severity, exacerbation patterns, and potential infectious triggers represents an essential component of diagnostic assessment.

Because the original PANDAS criteria require a temporal association with Group A Streptococcus (GAS) infection, evaluation typically includes microbiologic and serologic investigations. Throat culture remains the gold standard for identifying active pharyngeal GAS infection. Rapid antigen detection tests provide faster results but possess lower sensitivity. When suspicion remains high despite negative rapid testing, confirmatory culture is generally recommended [3].

Serologic studies commonly include measurement of antistreptolysin-O (ASO) and anti-DNase B titers. These antibodies serve as markers of prior streptococcal exposure rather than indicators of active infection [21-24]. Interpretation requires caution because elevated titers may persist for months following infection. Furthermore, substantial interindividual variability exists, and healthy children frequently demonstrate elevated antibody levels. Consequently, isolated elevations provide limited diagnostic value. Serial measurements demonstrating rising titers are generally considered more informative than single values [23,24].

Routine laboratory evaluation may additionally include complete blood count, inflammatory markers, metabolic testing, thyroid studies, autoimmune screening, and infectious disease investigations when clinically indicated. Such testing primarily serves to exclude alternative diagnoses rather than confirm PANDAS [44-46].

Neuroimaging is not routinely required for diagnosis. However, magnetic resonance imaging (MRI) may be appropriate when atypical neurologic findings, seizures, focal deficits, encephalopathic features, or concern for structural pathology are present. Although neuroimaging abnormalities have been reported in PANDAS cohorts, most patients demonstrate normal conventional MRI findings [32-34].

Similarly, lumbar puncture is not routinely indicated but may be warranted when autoimmune encephalitis, central nervous system infection, inflammatory neurologic disease, or other neuroimmunologic disorders are suspected. Cerebrospinal fluid analysis remains primarily a tool for differential diagnosis rather than confirmation of PANDAS [44-46].

Because no definitive diagnostic test exists, multidisciplinary evaluation frequently represents the most effective approach. Collaboration among pediatricians, psychiatrists, neurologists, immunologists, infectious disease specialists, psychologists, and educational professionals often improves diagnostic accuracy and facilitates comprehensive management [44,45].

Recent guidance documents have increasingly emphasized a balanced diagnostic approach: clinicians should recognize PANS/PANDAS as a potential clinical syndrome while also avoiding over attribution of common pediatric psychiatric presentations to infection-triggered autoimmunity [44,45]. The 2025 American Academy of Pediatrics clinical report acknowledges PANS as a likely valid diagnosis but stresses the absence of disease-specific biomarkers, the importance of differential diagnosis, and the need for multicenter studies [44]. Nordic clinical guidance and a 2024 Delphi consensus similarly emphasize structured evaluation, careful follow-up, and multidisciplinary management rather than reliance on any single laboratory test [45,47].

### 4.2 Diagnostic Challenges

Because no validated biomarker exists, diagnosis remains fundamentally clinical [44-46]. Clinicians must integrate symptom history, evidence of infectious exposure, psychiatric assessment, neurologic examination, laboratory findings, and exclusion of alternative diagnoses.

This process is complicated by the substantial overlap between PANDAS and other pediatric neuropsychiatric disorders. OCD, Tourette syndrome, autoimmune encephalitis, functional neurologic disorders, pediatric bipolar disorder, and anxiety disorders may all present with overlapping features [44-46].

Consequently, diagnostic uncertainty remains common. Current consensus recommendations emphasize comprehensive multidisciplinary assessment rather than reliance upon any single laboratory or clinical finding [44-46].

A practical diagnostic framework should therefore distinguish three levels of certainty: a clinical acute-onset neuropsychiatric syndrome, evidence of recent infection or immune activation, and evidence suggesting immune-mediated central nervous system involvement. Separating these levels may reduce both under recognition of legitimate neuroimmune disease and overdiagnosis of common psychiatric presentations [44,45].

### 4.3 Biomarker Controversy

The absence of validated biomarkers remains one of the greatest obstacles confronting PANDAS research. In most areas of medicine, diagnostic confidence is strengthened through objective laboratory testing, imaging findings, or histopathologic confirmation. In contrast, PANDAS remains fundamentally a clinical diagnosis [44-46].

Considerable effort has therefore been devoted to identifying biologic markers capable of confirming disease presence, predicting prognosis, or guiding treatment selection. Among these efforts, the development of antibody-based assays has generated both optimism and controversy [10].

The Cunningham Panel became one of the most widely discussed biomarker approaches in the field. Designed to measure antibodies against dopamine receptors, lysoganglioside GM1, tubulin, and CaMKII activation, the assay was intended to provide objective evidence of autoimmune neuropsychiatric disease [5].

Initial studies suggested potential utility. Investigators reported elevated antibody levels and abnormal CaMKII activation among children diagnosed with PANDAS and related disorders. These findings were interpreted as support for autoimmune pathophysiology and raised hopes that laboratory testing might facilitate diagnosis [5].

However, subsequent independent evaluations produced less encouraging results. Several studies failed to replicate previously reported diagnostic performance characteristics. Hesselmark and Bejerot demonstrated substantial overlap between presumed PANS/PANDAS patients and healthy controls, raising concerns regarding sensitivity, specificity, and reproducibility [10].

Methodological concerns further complicated interpretation. Many validation studies involved small sample sizes, referral-center populations, variable laboratory procedures, and inconsistent diagnostic criteria. Consequently, disagreement emerged regarding whether observed abnormalities reflected genuine disease mechanisms or methodological artifacts [10,12].

The Cunningham Panel controversy illustrates a broader neuroimmunology challenge: exploratory biomarkers may appear promising but fail to maintain diagnostic performance during independent validation [10,12].

At present, no biomarker possesses sufficient sensitivity or specificity to independently confirm or exclude PANDAS. Most consensus guidelines therefore continue to emphasize that diagnosis remains clinical and that laboratory findings should be interpreted within the broader clinical context [44-46].

An important possibility is that biomarker discovery has been hindered by the assumption that PANDAS or PANS represent a single disease entity. If current diagnostic frameworks capture several biologically distinct subgroups, then any individual antibody, cytokine, imaging marker, or immune-cell signature may perform poorly across the entire clinical population despite being meaningful within a narrower subgroup. This interpretation supports multimodal biomarker strategies that combine clinical phenotype, longitudinal course, immune profiling, neuroimaging, and treatment response rather than relying on a single stand-alone assay [10,30,41].

### 4.4 GAS Causality vs Carrier Status

One of the most important epidemiologic criticisms of PANDAS involves the prevalence of asymptomatic streptococcal carriage. Depending upon age, geography, and season, approximately 3–15% of healthy children may harbor GAS within the pharynx without evidence of active infection [23,24]. This carrier state creates substantial diagnostic uncertainty. A positive throat culture obtained during symptom exacerbation may simply reflect colonization rather than a causally relevant infection.

Consequently, demonstrating temporal association between GAS and symptom onset becomes considerably more difficult [21,23,24].

Similarly, elevated antistreptococcal antibody titers present interpretive challenges. Anti-streptolysin-O (ASO) and anti-DNase B titers frequently remain elevated for months following infection. Therefore, elevated titers may indicate remote exposure rather than recent infection temporally related to symptom development [21,23,24].

Because streptococcal infections are so common in childhood, coincidence remains a plausible explanation for at least some observed associations. This issue represents one of the strongest arguments advanced by critics of the PANDAS construct [9,20,21].

Resolving this challenge will likely require studies incorporating precise microbiologic characterization, serial antibody measurements, molecular strain typing, and longitudinal symptom tracking. Until such evidence becomes available, uncertainty regarding causality will persist [9,21,40].

### 4.5 Overdiagnosis and Medicalization Concerns

As public awareness of PANDAS has increased, concerns regarding overdiagnosis have emerged. Advocacy organizations, social media communities, online forums, and popular media have dramatically expanded public familiarity with the syndrome.

Although increased awareness may facilitate recognition of legitimate cases, it also raises the possibility of diagnostic inflation [9,44]. Symptoms commonly attributed to PANDAS—including anxiety, emotional lability, irritability, obsessive thoughts, sleep disturbance, and transient tic behaviors—occur frequently in childhood psychiatric disorders.

Critics argue that broad application of PANDAS terminology may encourage excessive medicalization of psychiatric symptoms [9]. Under this scenario, children with conventional OCD, anxiety disorders, or tic disorders may undergo extensive infectious and immunologic evaluations despite limited supporting evidence.

Potential consequences include delayed psychiatric treatment, unnecessary antibiotic exposure, financial burden, and increased family distress. These concerns underscore the importance of maintaining rigorous diagnostic standards and avoiding premature attribution of symptoms to autoimmune mechanisms [9,44].

At the same time, proponents argue that fear of overdiagnosis should not result in dismissal of genuinely immune-mediated disease. The challenge facing clinicians is therefore not choosing between psychiatric and immunologic explanations but rather determining when each framework is most appropriate.

## 5. Differential Diagnoses

Perhaps the greatest challenge in PANDAS evaluation involves distinguishing the syndrome from other neuropsychiatric and neurologic disorders that present with overlapping symptoms, as shown in Table 1, and discussed below.

### 5.1 Primary Obsessive-Compulsive Disorder

Primary OCD represents the most important differential diagnosis. Childhood OCD commonly involves contamination fears, checking rituals, intrusive thoughts, reassurance seeking, and repetitive behaviors. Distinguishing PANDAS from conventional OCD depends largely upon symptom trajectory. Whereas primary OCD generally develops gradually, PANDAS is characterized by abrupt onset and episodic exacerbations [3,8]. Nevertheless, symptom overlap remains substantial, and differentiating these conditions can be difficult in clinical practice.

### 5.2 Tourette Syndrome and Chronic Tic Disorders

Tourette syndrome and chronic tic disorders frequently coexist with OCD and may exhibit waxing-and-waning symptom courses. Because tic severity naturally fluctuates, apparent temporal relationships with infection may occasionally occur by chance. This overlap has contributed significantly to controversy surrounding PANDAS and underscores the importance of careful longitudinal assessment [9,26].

### 5.3 Sydenham Chorea

Sydenham chorea remains one of the most important neurologic differential diagnoses [1,2]. Like PANDAS, Sydenham chorea follows streptococcal infection and involves autoimmune-mediated basal ganglia dysfunction. However, choreiform movements are typically more prominent, and diagnosis often occurs within the broader context of rheumatic fever. Given the established autoimmune nature of Sydenham chorea, comparison between these conditions continues to inform mechanistic hypotheses regarding PANDAS [1,2].

### 5.4 Autoimmune Encephalitis

Increasing recognition of autoimmune encephalitis has significantly influenced contemporary neuropsychiatry. Anti-NMDA receptor encephalitis and related disorders may present with psychiatric symptoms, cognitive dysfunction, behavioral changes, movement abnormalities, seizures, and autonomic instability. Although these disorders differ substantially from PANDAS, overlap in early symptom presentation occasionally creates diagnostic uncertainty [48].

### 5.5 Systemic Autoimmune Disease

Neuropsychiatric manifestations may occur in systemic lupus erythematosus, vasculitis, antiphospholipid syndrome, and other autoimmune disorders. Appropriate laboratory screening should therefore be considered when clinical findings suggest broader systemic involvement.

### 5.6 Functional Neurologic Disorders and Mood Disorders

Functional neurologic disorders, pediatric bipolar disorder, severe anxiety disorders, trauma-related disorders, and developmental conditions may all present with behavioral changes, emotional dysregulation, and cognitive complaints. Comprehensive psychiatric assessment remains essential to avoid diagnostic error.

In practice, the absence of definitive biomarkers requires systematic evaluation of alternative explanations before attributing symptoms to PANDAS [44-46].

## 6. Therapeutic Management

### 6.1 General Principles

Management of suspected PANDAS requires a multidisciplinary approach that addresses psychiatric symptoms, infectious considerations, family functioning, educational support, and potential immune dysregulation. Importantly, treatment should be individualized because disease severity, symptom patterns, and biologic mechanisms likely vary considerably among patients [44-46].

The current evidence base remains limited, and no universally accepted treatment algorithm exists. Consequently, therapeutic decisions frequently involve balancing potential benefits against uncertainty regarding efficacy [12,40].

A 2021 systematic review of anti-inflammatory, antibacterial, and immunomodulatory treatments concluded that existing studies suggest possible benefit in selected patients but remain limited by small samples, heterogeneity, and risk of bias. This conclusion remains important for interpretation of later open-label IVIG studies: promising improvements should be considered hypothesis-strengthening rather than definitive proof of efficacy until adequately powered randomized trials are completed [40].

Therapeutic evidence should also be interpreted according to treatment target. Antibiotics primarily address confirmed or suspected infection; CBT and SSRIs address obsessive-compulsive and anxiety symptoms regardless of etiology; and anti-inflammatory or immunomodulatory therapies attempt to modify presumed immune mechanisms. Conflating these targets can lead to overstated conclusions about efficacy or mechanism [44,45].

### 6.2 Antibiotic Therapy

Treatment of documented GAS infection remains standard, using antibiotics consistent with infectious disease guidelines [23,44].

The rationale for antibiotic treatment extends beyond eradication of infection. If streptococcal exposure contributes directly to symptom exacerbation, prompt treatment might theoretically reduce immune stimulation and prevent disease progression. Several studies have reported symptomatic improvement following antimicrobial therapy, particularly among patients with documented recent infection [24,48,49].

Randomized investigations have produced mixed findings. Pilot studies involving cefdinir and azithromycin demonstrated encouraging trends toward symptom improvement, although sample sizes were small and statistical power was limited. Consequently, while available data support treatment of active infection, evidence supporting antibiotics as primary neuropsychiatric therapy remains incomplete [48,49].

The antibiotic literature illustrates the broader causality problem in PANDAS research. Clinical improvement after antibiotics may reflect eradication of active GAS infection, anti-inflammatory properties of some agents, natural symptom fluctuation, placebo effects, or concurrent psychiatric treatment. Trials that distinguish active infection, carrier state, and postinfectious immune activity are needed before antibiotics can be interpreted as disease-modifying neuropsychiatric therapy [21,23,24].

### 6.3 Prophylactic Antibiotics

Prophylactic antibiotics remain controversial. Some studies report fewer streptococcal infections and exacerbations, but concerns about resistance, adverse effects, and limited evidence have prevented broad adoption [23,40,50].

Current consensus recommendations generally reserve prophylactic strategies for carefully selected patients after multidisciplinary evaluation [44,45].

### 6.4 Cognitive Behavioral Therapy and Psychiatric Management

Regardless of etiology, obsessive-compulsive symptoms remain among the most disabling manifestations of PANDAS. Consequently, evidence-based psychiatric treatment remains essential.

Cognitive behavioral therapy (CBT), particularly exposure and response prevention (ERP), remains a central psychiatric intervention for OCD symptoms in PANS/PANDAS management [51,52].

Importantly, CBT addresses symptom expression regardless of underlying cause. Even if immune-mediated processes contribute to symptom development, patients may benefit from behavioral interventions targeting compulsive behaviors and maladaptive anxiety responses [44,51,52].

Family involvement frequently enhances treatment effectiveness because caregivers often become inadvertently incorporated into obsessive-compulsive rituals. Psychoeducation and family-based interventions therefore play important supportive roles. [51,52]

### 6.5 Selective Serotonin Reuptake Inhibitors

Selective serotonin reuptake inhibitors (SSRIs) remain standard pharmacologic treatment for pediatric OCD symptoms, including when such symptoms occur in PANS/PANDAS populations [15,51]. However, several investigators have suggested that children with PANDAS may exhibit increased sensitivity to psychotropic medications. Reports of behavioral activation, irritability, emotional lability, agitation, and worsening symptoms have prompted recommendations favoring cautious dosing and gradual titration [51,53].

Despite these concerns, SSRIs remain an important component of treatment for many patients. Their use should not be delayed solely because symptoms are suspected to have an immune-mediated component [44,46].

From a clinical standpoint, psychiatric and immunologic approaches should be viewed as complementary rather than mutually exclusive. Even when immune activation contributes to symptom onset, entrenched compulsive behaviors, avoidance patterns, family accommodation, and school disruption often require evidence-based psychiatric and behavioral intervention. This integrated model may reduce false dichotomies between medical and psychiatric care.

### 6.6 Nonsteroidal Anti-Inflammatory Drugs

Because neuroinflammation has been implicated in disease pathogenesis, NSAIDs have attracted interest as potential adjunctive therapies. Observational studies suggest that NSAID administration during symptom exacerbations may reduce flare duration and symptom severity in some patients. [54]

The biological rationale is plausible but incomplete. By suppressing inflammatory signaling pathways, NSAIDs may theoretically mitigate immune-mediated neural dysfunction. Nevertheless, evidence remains largely observational, and controlled prospective studies remain limited [40,54].

Consequently, NSAIDs are generally considered low-risk adjunctive therapies rather than definitive disease-modifying treatments.

### 6.7 Corticosteroids

Corticosteroids represent one of the most frequently utilized immunomodulatory interventions in PANS/PANDAS populations. Their potent anti-inflammatory effects provide a strong theoretical rationale for use in immune-mediated neuropsychiatric disease [36,55].

Observational studies have reported reductions in symptom duration and severity following corticosteroid administration. Some patients demonstrate rapid improvement, further fueling interest in immune-mediated mechanisms [55].

However, corticosteroids are associated with significant adverse effects, including mood changes, irritability, anxiety, insomnia, weight gain, hypertension, metabolic disturbances, and immunosuppression. Notably, neuropsychiatric side effects may paradoxically worsen behavioral symptoms.

For these reasons, corticosteroids are generally reserved for carefully selected patients experiencing severe symptom exacerbations [36,44,45].

### 6.8 Intravenous Immunoglobulin

Intravenous immunoglobulin (IVIG) remains among the most controversial treatments in the PANDAS literature. IVIG exerts complex immunomodulatory effects and has demonstrated efficacy across numerous autoimmune neurologic disorders [35-37,40].

Early randomized investigations reported significant improvement in obsessive-compulsive symptoms following IVIG administration. Subsequent observational studies have described similar findings in selected patients [35,36].

Recent prospective and open-label IVIG studies have added important but still nondefinitive evidence. Hajjari and colleagues reported broad clinical improvement after three monthly IVIG treatments in a small prospective open-label cohort, while Eremija and colleagues reported improvements in multiple neuropsychiatric and cognitive domains after IVIG treatment. A 2024 open-label immunophenotyping study found that IVIG response correlated with reductions in pro-inflammatory monocytes and improvements in neuropsychiatric measures. These studies support continued investigation of immunomodulation but do not eliminate the need for larger blinded trials because placebo effects, regression to the mean, and natural fluctuation remain major interpretive concerns [37-39,56].

Nevertheless, evidence remains limited by small sample sizes, inconsistent replication, heterogeneous patient populations, and methodological limitations. Systematic reviews generally conclude that IVIG may benefit some patients, but that current evidence remains insufficient to support routine use [40].

Given cost and resource intensity, guidelines generally reserve IVIG for moderate-to-severe cases after specialist evaluation [44,45].

### 6.9 Plasma Exchange

Plasma exchange represents the most aggressive immunomodulatory therapy utilized in PANDAS. The procedure removes circulating antibodies and immune mediators from the bloodstream and has established efficacy in several antibody-mediated neurologic disorders [35,36].

Early clinical trials reported dramatic improvement in both obsessive-compulsive symptoms and tic severity among severely affected children. These findings provided some of the strongest indirect evidence supporting autoimmune mechanisms [35,36].

However, plasma exchange carries substantial procedural risks and requires specialized expertise. Consequently, it is generally reserved for exceptionally severe, treatment-resistant presentations [44,45].

## 7. Immunotherapy Controversies

Immunotherapy remains one of the most debated aspects of PANDAS/PANS management. Advocates argue that dramatic responses observed in selected patients provide compelling evidence for immune-mediated disease. Critics counter that much of the literature relies upon uncontrolled studies vulnerable to placebo effects, regression to the mean, referral bias, and diagnostic uncertainty [12,40].

A balanced interpretation is warranted. Available evidence suggests that some children may indeed experience clinically meaningful benefit from immunomodulatory interventions. [37-40,56] However, current data remain insufficient to identify which patients are most likely to respond or to justify widespread use of aggressive therapies [12,40].

Future precision-medicine approaches incorporating immunophenotyping, biomarkers, and objective outcome measures may ultimately help resolve these controversies [30,39,41].

The apparent discrepancy between compelling individual clinical responses and limited randomized-trial evidence represents one of the central challenges in the PANDAS literature. Several explanations are plausible. First, immune-responsive patients may represent only a subset of individuals currently classified under the broader PANDAS or PANS framework, thereby diluting treatment effects in heterogeneous study populations. Second, the relapsing-remitting nature of symptom trajectories increases susceptibility to regression-to-the-mean effects. Third, placebo responses may be particularly influential in disorders characterized by fluctuating symptom severity and high caregiver investment. Consequently, future immunotherapy trials will likely require biomarker-guided patient selection rather than reliance upon symptom-based diagnostic criteria alone [30,39,41].

### 7.1 Long-Term Outcomes and Prognosis

Long-term outcomes in PANDAS and PANS remain incompletely characterized because prospective studies following children into adolescence and adulthood are limited. Available evidence suggests a heterogeneous course rather than a single prognosis. Some children experience substantial remission, others follow a relapsing-remitting pattern, and a subset develop persistent psychiatric symptoms or functional impairment [8,43].

Relapses are commonly described after infectious illnesses, particularly GAS, but exacerbations may also follow non-streptococcal infections, psychosocial stressors, or no identifiable trigger. The strength of the infection-relapse relationship varies across cohorts, likely reflecting differences in diagnostic criteria, microbiologic confirmation, treatment exposure, and follow-up duration [9,19-21].

Remission is possible and may be substantial, especially when symptoms are recognized early and managed with coordinated psychiatric, educational, infectious disease, and immunologic input. However, symptom reduction does not always mean full functional recovery. Residual anxiety, compulsive behaviors, tics, attentional problems, school avoidance, or family accommodation may persist after acute symptoms improve [8,43].

Adult outcomes are particularly uncertain. Some patients appear to improve during adolescence, whereas others continue to experience chronic OCD, tic symptoms, anxiety, mood symptoms, or functional impairment. Because few cohorts extend into adulthood, it remains unclear whether PANDAS is usually self-limited, a relapsing-remitting neuroimmune condition, or a risk factor for later psychiatric morbidity in biologically susceptible subgroups [9,12,41].

Functional outcomes deserve greater emphasis than symptom counts alone. Abrupt neuropsychiatric deterioration can disrupt school attendance, peer relationships, family routines, and caregiver well-being. Families may face substantial emotional, logistical, and financial burden, particularly when symptoms are severe, relapsing, or diagnostically uncertain [44].

Overall, current evidence supports cautious optimism while underscoring prognostic uncertainty. Future studies should use standardized symptom scales, school-function measures, quality-of-life instruments, caregiver-burden assessments, and long-term follow-up to identify predictors of relapse, remission, functional recovery, and adult outcome [41,44].

### 7.2 Major Controversies in PANDAS Research

More than twenty-five years after its original description, PANDAS remains among the most controversial diagnoses in pediatric neuropsychiatry. Unlike many disputed medical conditions that lack biologic plausibility, debate surrounding PANDAS persists despite the presence of substantial immunologic, epidemiologic, and translational evidence [1,31]. The controversy therefore centers not on whether immune-mediated neuropsychiatric disease can occur, but rather on how frequently it occurs, how it should be diagnosed, and which patients truly belong within the PANDAS construct [10,40].

Guideline disagreement has itself become part of the contemporary controversy. The 2025 AAP clinical report places strong emphasis on cautious diagnosis, limited evidence for many interventions, and the need for additional multicenter research [44]. By contrast, Nordic guidance and Delphi-based publications tend to emphasize broader multidisciplinary evaluation and careful recognition of inflammatory or immune-mediated subgroups [45,47]. These perspectives are not entirely incompatible: together, they indicate that clinicians should neither dismiss abrupt-onset neuropsychiatric deterioration nor assume that every such presentation reflects PANDAS/PANS [44,45,47].

### 7.3 Controversy 1: Is PANDAS a Distinct Clinical Entity?

A fundamental unresolved question is whether PANDAS represents a discrete disease entity or a descriptive clinical phenotype. Supporters argue that the syndrome demonstrates sufficient consistency in symptom onset, temporal relationship to infection, neurologic findings, and treatment response to justify recognition as a unique disorder [1,3].

Critics counter that many defining characteristics of PANDAS are nonspecific. Obsessive-compulsive symptoms, tic disorders, anxiety, emotional lability, and behavioral dysregulation occur frequently in childhood psychiatric populations independent of infectious triggers. Because symptom overlap is extensive, some investigators argue that PANDAS may simply represent one manifestation within a broader spectrum of childhood neuropsychiatric illness [9,12].

An emerging perspective attempts to reconcile these positions by viewing PANDAS as a biologically heterogeneous syndrome. Under this model, infection-triggered autoimmunity may contribute substantially to symptom development in some patients while playing little or no role in others. This framework acknowledges both the existence of genuine neuroimmune disease and the limitations of current diagnostic criteria [31,41].

### 7.4 Controversy 2: The Biomarker Problem

The absence of validated biomarkers remains perhaps the single greatest obstacle to scientific progress. Most medical disorders ultimately become easier to study once objective diagnostic tests are developed. In contrast, PANDAS diagnosis continues to rely primarily upon clinical judgment [10,44-46].

The Cunningham Panel controversy exemplifies this challenge [10]. Initial investigations generated enthusiasm by suggesting that antineuronal antibodies and CaMKII activation might provide objective evidence of autoimmune neuropsychiatric disease [5]. However, independent validation studies failed to consistently replicate these findings [10].

Importantly, failure of a particular biomarker does not necessarily invalidate the underlying disease hypothesis. Many neurologic and autoimmune disorders required decades of research before reliable biomarkers emerged. Nevertheless, the absence of validated laboratory testing continues to limit diagnostic precision and contributes substantially to ongoing skepticism [10,44].

Future biomarker development will likely require integration of multiple biologic domains rather than reliance upon single laboratory assays. Composite approaches incorporating immune markers, imaging findings, genomics, and clinical features may ultimately prove more successful than antibody testing alone [41,44].

### 7.5 Controversy 3: Overdiagnosis and Diagnostic Expansion

Increasing awareness of PANDAS has produced both benefits and challenges. Greater recognition may facilitate identification of children with potentially treatable neuroimmune disorders. At the same time, concerns regarding overdiagnosis have become increasingly prominent [9,44].

Many symptoms associated with PANDAS—including anxiety, emotional dysregulation, obsessive thoughts, sensory sensitivities, sleep disturbances, and transient tics—occur commonly in pediatric psychiatric populations. Consequently, broad application of the diagnosis may result in attribution of nonspecific symptoms to immune mechanisms despite limited supporting evidence [9].

The evolution from PANDAS to PANS has further complicated this issue. While the broader PANS framework may improve sensitivity for identifying affected patients, it may simultaneously reduce diagnostic specificity. As diagnostic boundaries expand, distinguishing true neuroimmune disease from conventional psychiatric illness becomes increasingly difficult [9,11,41].

Maintaining rigorous diagnostic standards therefore remains essential for both clinical practice and research.

### 7.6 Controversy 4: Immunotherapy Skepticism

The use of immunomodulatory therapies represents one of the most polarizing issues in the field. Proponents cite dramatic clinical responses observed following corticosteroids, intravenous immunoglobulin (IVIG), and plasma exchange as evidence supporting autoimmune mechanisms [35-39,55,56].

Critics emphasize that much of the treatment literature relies upon small sample sizes, observational designs, and subjective outcome measures. Placebo effects, regression to the mean, natural symptom fluctuation, and selection bias may contribute significantly to observed improvements [12,40].

The reality is likely nuanced. Available evidence suggests that some patients experience meaningful benefit from immunotherapy [37-40,56]. However, current data remain insufficient to define optimal treatment algorithms or identify the patients most likely to respond.

Future randomized controlled trials incorporating standardized diagnostic criteria and objective outcome measures will be necessary to clarify the role of immunomodulatory interventions [40,41].

### 7.7 Controversy 5: Referral Bias, Selection Bias, and Limited Reproducibility

Another major methodological concern involves referral bias. Much of the published literature on PANDAS originates from highly specialized academic centers and tertiary referral clinics [9,12]. Patients referred to these centers often represent unusually severe, treatment-resistant, or medically complex cases.

As a result, findings derived from specialty clinics may not accurately reflect the broader population of children presenting with OCD, tic disorders, or acute neuropsychiatric symptoms. Referral bias can artificially inflate estimates of disease severity, treatment responsiveness, and prevalence of autoimmune abnormalities [9,12].

Selection bias may similarly influence immunologic and neuroimaging investigations. Studies frequently recruit participants who already exhibit clinical features suggestive of immune dysfunction, increasing the likelihood of identifying biologic abnormalities. Such approaches may inadvertently overestimate associations between immune markers and psychiatric symptoms [10,12].

### 7.8 Why the Controversy Persists

The controversy persists because current criteria identify a clinical syndrome rather than a biologically validated disease; biomarkers are not reproducible enough for diagnosis; cohorts are heterogeneous; psychiatric overlap complicates causal inference; and treatment studies remain small and methodologically variable. These factors explain why compelling individual cases coexist with inconsistent population-level evidence [9,10,12,40,41].

## 8. Recent Literature Updates (2021–2026)

The 2021–2026 literature meaningfully strengthens several parts of the manuscript while preserving the need for caution. Updated clinical guidance from Nordic experts, an Italian Delphi consensus, and the American Academy of Pediatrics supports careful recognition of PANS/PANDAS while emphasizing multidisciplinary assessment and diagnostic uncertainty [44,45,47]. Systematic review evidence continues to characterize treatment studies as promising but methodologically limited. [40] Recent neuroimaging and immunology studies suggest that basal ganglia dysfunction and immune abnormalities remain biologically plausible contributors to disease pathophysiology [30,34,39]. New cohort evidence also supports the possibility that some PANS patients have broader immune dysregulation or subsequent autoimmune disease risk [18]. Finally, recent IVIG studies suggest clinical and immunologic improvement in selected patients but remain primarily open-label and require confirmation in larger randomized trials [37-39,56].

Taken together, recent literature does not resolve the controversy but reinforces the likelihood of heterogeneous subgroups, some infection-triggered or immune-mediated. The most defensible position is therefore a precision-medicine framework focused on reproducible clinical, immunologic, imaging, and longitudinal features.

## 9. Limitations of Current Evidence

Within the PANDAS literature, reproducibility remains a persistent challenge [10,40]. Several factors contribute to this problem: small sample sizes, referral-center recruitment, variable diagnostic criteria, inconsistent laboratory methodology, heterogeneous outcome measures, lack of blinded assessment, and limited longitudinal follow-up. These issues complicate interpretation of both positive and negative findings. Importantly, many apparent contradictions within the literature may reflect methodological differences rather than true biologic disagreement [9,10,12].

Interpretation of the PANDAS/PANS literature is constrained by several recurring methodological limitations. Many studies are small, originate from specialty referral centers, and enroll patients with severe or diagnostically complex presentations. These factors limit generalizability and may inflate estimates of disease severity, immune abnormalities, and treatment responsiveness [9,12].

Diagnostic heterogeneity is a central problem. Studies vary in how they define PANDAS, PANS, acute-onset OCD, tic disorders, infectious exposure, symptom exacerbation, and clinical severity. Methods for confirming GAS infection also differ, and many investigations rely on single throat cultures, serologic titers, administrative codes, or retrospective histories. These differences complicate direct comparison across cohorts [9,20,21].

Biomarker and treatment studies face additional challenges. Proposed immune markers, including antineuronal antibodies, CaMKII activation, cytokine profiles, and imaging abnormalities, have not demonstrated sufficient reproducibility for routine diagnosis [10,29]. Treatment evidence is limited by open-label designs, small samples, variable outcome measures, placebo effects, regression to the mean, and natural symptom fluctuation [12,40].

Finally, long-term outcome data remain sparse. Few studies follow patients into adulthood or assess school functioning, quality of life, caregiver burden, and relapse patterns with standardized measures. These limitations do not negate the biologic plausibility of infection-triggered neuroimmune syndromes, but they do require cautious interpretation and highlight the need for rigorous multicenter prospective research.

## 10. Future Directions

Future progress will depend on moving from symptom-based labels toward biologically informed classification. The highest priority is development of prospective multicenter cohorts using standardized diagnostic criteria, uniform infectious testing, blinded assessments, validated outcome measures, and longitudinal follow-up across acute illness, remission, relapse, and recovery.

Precision immunophenotyping should be central to this effort. Longitudinal studies measuring autoantibodies, cytokines, immune-cell subsets, complement activation, and inflammatory signaling may help distinguish causal mechanisms from nonspecific immune activation or consequences of severe psychiatric stress.

Neuroimaging research should shift from isolated structural findings toward multimodal models that integrate diffusion imaging, functional connectivity, inflammatory imaging, symptom trajectories, and immune biomarkers. Such approaches may clarify whether basal ganglia and cortico-striato-thalamo-cortical abnormalities define biologically meaningful subgroups or represent nonspecific correlates of pediatric neuropsychiatric illness.

Genomic, microbiome, and cerebrospinal fluid studies may further refine patient stratification. Genetic susceptibility, blood-brain barrier biology, host-microbiome interactions, and central nervous system immune signatures remain plausible but underdeveloped areas of investigation.

Computational and machine-learning approaches may eventually integrate clinical phenotype, infection history, laboratory data, neuroimaging, genetics, treatment response, and longitudinal course to improve classification and treatment selection. These tools will be useful only if trained on large, well-characterized, externally validated datasets.

Therapeutic trials should increasingly use biomarker-informed enrollment, blinded outcome assessment, standardized psychiatric co-interventions, and longer follow-up. The goal should not be to prove that one treatment applies to all patients labeled PANDAS/PANS, but to identify which interventions benefit which biologically defined subgroups and at what stage of illness.

## 11. Conclusion

More than a quarter century after its initial description, Pediatric Autoimmune Neuropsychiatric Disorders Associated with Streptococcal Infections (PANDAS) remains a scientifically important but contested construct in pediatric neuropsychiatry. The evidence reviewed here supports the biologic plausibility of infection-triggered neuroimmune mechanisms contributing to abrupt-onset obsessive-compulsive symptoms, tic disorders, and related neuropsychiatric manifestations in at least a subset of children. Parallels with Sydenham chorea, epidemiologic associations with streptococcal exposure, antineuronal antibody studies, basal ganglia and network-level neuroimaging findings, translational animal models, and selective responses to immunomodulatory therapies collectively indicate that immune-mediated neuropsychiatric disease can occur in pediatric populations [1,8,31].

At the same time, the current evidence base does not support a single uniform disease model for all patients meeting PANDAS or PANS criteria. Diagnostic definitions vary across studies, validated biomarkers remain unavailable, and findings from antibody, epidemiologic, neuroimaging, and treatment investigations have not been consistently replicated. Small sample sizes, referral-center recruitment, retrospective designs, inconsistent outcome measures, and limited long-term follow-up further constrain causal inference. Accordingly, neither uncritical acceptance nor categorical rejection of the PANDAS construct is justified by the available literature [10,40].

The most defensible interpretation is that PANDAS and related acute-onset neuropsychiatric presentations likely represent biologically heterogeneous conditions rather than a single discrete disorder. Infection-triggered or immune-mediated mechanisms may be central in some patients, contributory in others, and absent in cases better explained by primary psychiatric, neurodevelopmental, or functional disorders. This heterogeneity provides a plausible explanation for inconsistent biomarker performance, variable treatment responsiveness, and divergent long-term outcomes.

Clinically, the evidence supports a balanced multidisciplinary approach. Children with abrupt-onset OCD, tics, restrictive eating, neurologic abnormalities, or severe functional decline warrant careful evaluation for infectious, inflammatory, neurologic, and psychiatric contributors. At the same time, clinicians should avoid over attribution of common pediatric psychiatric symptoms to autoimmune disease in the absence of supportive clinical features. Evidence-based psychiatric care remains essential, while antimicrobial and immunomodulatory treatments should be individualized according to documented infection, illness severity, comorbidity, risk-benefit considerations, and specialist input [44,45,51,52].

The central task for the field is to move beyond debate over diagnostic labels and toward a precision-medicine framework for childhood neuroimmune neuropsychiatry. Rigorous prospective multicenter studies, standardized diagnostic criteria, validated outcome measures, longitudinal follow-up, and integration of immunophenotyping, genomics, neuroimaging, microbiome science, and computational modeling will be necessary to clarify mechanisms and identify immune-responsive subgroups. Such work has the potential not only to refine the PANDAS/PANS construct but also to advance broader understanding of how infection, immunity, and brain development interact to shape pediatric neuropsychiatric disease.

## Figures and Tables

**Table 1:** An overview of the differential diagnosis of PANDAS to distinguish the syndrome from other neuropsychiatric and neurologic disorders that present with overlapping symptoms.

	Primary Obsessive- Compulsive Disorder	Tourette Syndrome & Chronic Tic Disorders	Sydenham Chorea	Autoimmune Encephalitis	Functional Neurologic Disorders	Systemic Autoimmune Diseases
**Key Features**	Obsessions & compulsions Rituals, intrusive thoughts, reassurance-seeking	Motor and/or vocal tics Premonitory urges	Involuntary, irregular choreiform movements Emotional lability	Psychiatric and cognitive dysfunction Seizures, movement abnormalities, autonomic instability	Emotional and behavioral dysregulation Neurologic symptoms inconsistent with recognized disease	Variable systemic symptoms (e.g., fever, rash, arthralgia) Variable neuropsychiatric involvement
**Onset & Course**	Insidious onset during childhood/early adolescence Often chronic	Childhood onset, waxing-and-waning May resolve during adolescence or persist into adulthood	Following group A streptococcal infection (1-6 months) Monophasic or relapsing	Subacute progressive onset (days-weeks) Monophasic or relapsing	Variable onset May be triggered or exacerbated by psychosocial stressors	Variable onset, prevalence increases with age Often relapsing-remitting or chronic
**Common Associated Findings**	Family history of OCD and/or anxiety disorders	Comorbid OCD and/or ADHD Family history of tics	Recent GAS infection Elevated ASO and/or anti-DNase B titers Systemic findings characterized by acute rheumatic fever (e.g., carditis, arthritis)	Presence of autoantibodies (e.g., anti-NMDA receptor) CSF pleocytosis or oligoclonal bands MRI and/or EEG abnormalities	Comorbid psychiatric disorders (e.g., anxiety, depression, PTSD) Normal physical exam, labs, and imaging	Presence of autoantibodies (e.g., ANA, antiphospholipid)
**Distinguishing Aspects from PANDAS**	Gradual, insidious onset with stable, non-episodic course No history of preceding infection or signs of immunologic response	No history of preceding infection or signs of immunologic response	Prominent chorea Association with acute rheumatic fever	Altered mental status, seizures, autonomic instability Positive inflammatory and autoimmune markers MRI and/or EEG abnormalities	No history of provoking infection or signs of immunologic response	Evidence of multiple organ system involvement Positive inflammatory and autoimmune markers
**Degree of Overlap with PANDAS**	Significant OCD symptoms are a core presentation of PANDAS	Significant Tics are a core presentation in PANDAS	Significant Shared infectious trigger and molecular mimicry etiology	Moderate Autoimmune etiology with psychiatric and movement symptoms	Moderate May share certain behavioral and emotional symptoms	Variable (generally low) Dependent on neuropsychiatric involvement

## 12. Key Points:

- PANDAS is a clinically recognized syndrome characterized by the abrupt onset of obsessive-compulsive symptoms and/or tic disorders temporally associated with Group A streptococcal infection, but its precise diagnostic boundaries and biologic validity remain incompletely defined.
- Current evidence supports biologically plausible immune-mediated mechanisms, including as molecular mimicry, antineuronal antibodies, neuroinflammation, blood-brain barrier dysfunction, and basal ganglia involvement, in a subset of affected children, although no single mechanism has been conclusively established.
- Although neuroimaging, immunologic, translational animal, and epidemiologic studies collectively support the concept of infection-triggered neuroimmune dysfunction, inconsistent replication and methodological heterogeneity continue to limit definitive causal inference.
- No validated biomarker currently possesses sufficient sensitivity or specificity for routine clinical diagnosis, making PANDAS a diagnosis based primarily on clinical assessment and careful exclusion of alternative neurologic and psychiatric disorders.
- Evidence-based psychiatric management such as cognitive behavioral therapy with exposure and response prevention and appropriately titrated SSRIs remains a cornerstone of treatment regardless of the suspected underlying immune mechanism.
- Immunomodulatory therapies, including corticosteroids, intravenous immunoglobulin, and plasma exchange, may benefit carefully selected patients with moderate-to-severe disease, but current evidence remains insufficient to support their routine use outside multidisciplinary specialist evaluation.
- Long-term outcomes are heterogeneous, ranging from complete remission to relapsing-remitting illness or persistent psychiatric morbidity, underscoring the importance of individualized management and long-term follow-up.
- The principal controversies surrounding PANDAS involve diagnostic specificity, biomarker reproducibility, causal attribution of streptococcal infection, potential overdiagnosis, and interpretation of immunotherapy studies rather than the existence of postinfectious neuroimmune disease itself.
- Future progress will depend on large prospective multicenter studies integrating standardized diagnostic criteria, longitudinal clinical phenotyping, advanced immunologic profiling, neuroimaging, genomics, and precision-medicine approaches to identify biologically meaningful patient subgroups.
